# Percolation Transitions in Edge-Coupled Interdependent Networks with Reinforced Inter-Layer Links

**DOI:** 10.3390/e26080693

**Published:** 2024-08-16

**Authors:** Junjie Zhang, Caixia Liu, Shuxin Liu, Kai Wang, Weifei Zang

**Affiliations:** 1 Institute of Information Technology, PLA Strategic Support Force Information Engineering University, Zhengzhou 450001, China; jj961004@stu.xjtu.edu.cn (J.Z.); wangkai0508@126.com (K.W.); 2 Institute of System Engineering, Academy of Military Sciences, Beijing 100091, China; 3 Department of Information Systems Security, PLA Information Engineering University, Zhengzhou 450001, China; zwfndsc@163.com

**Keywords:** robustness, phase transitions, edge-coupled interdependent networks, reinforced

## Abstract

Prior research on cascading failures within interdependent networks has predominantly emphasized the coupling of nodes. Nevertheless, in practical networks, interactions often exist not just through the nodes themselves but also via the connections (edges) linking them, a configuration referred to as edge-coupled interdependent networks. Past research has shown that introducing a certain percentage of reinforced nodes or connecting edges can prevent catastrophic network collapses. However, the effect of reinforced inter-layer links in edge-coupled interdependent networks has yet to be addressed. Here, we develop a theoretical framework for studying percolation models in edge-coupled interdependent networks by introducing a proportion of reinforced inter-layer links and deriving detailed expressions for the giant and finite components and the percolation phase transition threshold. We find that there exists a required minimum proportion of the reinforced inter-layer links to prevent abrupt network collapse, which serves as a boundary to distinguish different phase transition types of a network. We provide both analytical and numerical solutions for random and scale-free networks, demonstrating that the proposed method exhibits superior reinforcement efficiency compared to intra-layer link reinforcement strategies. Theoretical analysis, simulation results, and real network systems validate our model and indicate that introducing a specific proportion of reinforced inter-layer links can prevent abrupt system failure and enhance network robustness in edge-coupled interdependent networks.

## 1. Introduction

Complex networks provide powerful tools for modeling and studying various systems, such as the human brain, cyber-physical systems (CPSs), infrastructure networks, social networks [1,2,3], etc. Still, these systems usually do not operate in isolation but rather interact and cooperate, known as interdependent networks [4,5,6,7]. These systems are subject to internal local failures and external attacks or disturbances. Because of their interactions and interdependencies, the propagation of failures in the network can make the whole system more vulnerable and even lead to network dysfunction or disintegration, such as the blackouts in Italy in 2003, North America in 2008, Venezuela in 2019, and the global outbreak of COVID-19 in 2020 [4,8,9]. Therefore, research on the robustness of multilayer interdependent networks has attracted the attention of many scholars.

Classical percolation models, including bond percolation [10,11] or site percolation [12,13], have been instrumental in the study of the emergence of collective behavior in many systems [14,15]. These models have significantly advanced our understanding of the formation of giant connected components and phase transitions within network systems. Over the recent decade or so, the robustness of interdependent networks has been extensively studied using percolation theory. The interconnected nature of complex networks allows them to exhibit rich phase transition behavior, and the nature of these transitions usually depends on the structural characteristics of the network. For example, Parshani et al. [7] further proposed a partially interdependent network model based on the original fully interdependent network proposed by Buldyrev et al. [4]. They found that the strength of dependence can alter the behavior in the network. There are also specific topological and inter-layer coupling properties such as "weak" interdependence [16] and multiple dependence [17], which change the type of phase transitions in the network, thus increasing the robustness of the interdependent networks.

Nonetheless, the majority of studies have primarily focused on node-coupled interdependent networks (NINs), where interdependencies exist between nodes of two sub-networks, leading to the propagation of failures between these nodes. However, in many real-world networks, interactions often occur at the edges connecting nodes, rather than at the nodes themselves. Such configurations are referred to as edge-coupled interdependent networks (EINs). For example, in fiber and resource management networks, the transmission of optical signals from fiber links depends on the command and control of information transmission from links in the resource management network. Failure of a fiber link in the fiber network will result in no information being transmitted in the resource management network and vice versa. Similarly, the logistics and information networks form an interdependent relationship in the supply chain network. Therefore, the study of EINs has received considerable attention from experts and scholars [18,19,20,21,22].

The theoretical framework for analyzing EINs was first developed by Gao et al. and analyzed using the self-consistent probabilities equations [19]. The model is analyzed based on the bond percolation model since the coupling relationship exists between connected edges, and the results show that EINs are more robust than NINs, which can be reflected by the discontinuous phase transition threshold. On this basis, they introduced a partially coupled EIN model and showed how a change in the coupling strength *q* triggers a change in the type of phase transition in the network when only a proportion of edges depend on the edges in another network. Gao et al. also proposed an EIN model with directed dependency links (EINDDL) [23]. They found the effects of different proportions of connectivity links β on the phase transition behavior in the sub-network. In addition, inspired by node reinforcement [24], Zhang et al. introduced reinforced edges in EIN networks [25]. They observed the transition-free phenomenon of FC in the network, proving that introducing a certain proportion of reinforced intra-layer links in the network can effectively prevent the catastrophic collapse of the network.

To further improve network robustness and prevent the catastrophic collapse of the network, many methods such as a node or link reinforced method [24,26,27], disaster recovery method [28,29,30], and edge rewiring or addition have been proposed, one after another [31,32,33,34]. Among them, unlike the conventional assumption that only giant components survive, the reinforced method considers many realistic scenarios in which independently operating components exist in the network. A typical example illustrates this point. For instance, in an interdependent network consisting of a fiber-optic network and a resource management system, if a fiber-optic line fails, the service can be maintained by subsystems that can operate independently. Compared to other schemes, the protection of essential nodes in the network has attracted the attention of many experts and scholars due to the simplicity of implementation and the possibility of pre-emptive defense, e.g., Huang et al. devised strategies such as the protection of nodes with higher degrees [35]. Xie et al. established a theoretical framework to increase the robustness of interdependent networks by reinforcing a fraction of nodes [36]. However, the above strategies help improve a single network’s robustness. Still, they are less effective for interdependent networks because they need to consider the coupled interactions between networks fully.

Since the regular operation of two interacting system components (in terms of dependency links) in an EIN is essential to maintain the network’s functionality, and if reinforced measures (e.g., backups, etc.) are only performed in the sub-network, failures will also cause the network to collapse due to propagation through dependency links [26]. Therefore, it is necessary to consider dependency link-reinforced methods to improve the robustness of interdependent networks. Therefore, in this paper, we propose an EIN model with reinforced inter-layer links, where some dependency links are randomly selected as “reinforced links”. Then, the edges at both ends of the selected links are reinforced simultaneously. We then develop a theoretical framework for analyzing the model under random attacks based on the self-consistent probabilistic approach and on generating functions. Theoretical analyses and computer simulations of Erdős–Rényi (ER) [37] and scale-free (SF) [38] networks are carried out, and the results from real network datasets are simulated to investigate the relationship between the proportion of reinforced inter-layer links and the phase transition behaviors of the network. Our theoretical and numerical results show that the introduction of reinforced inter-layer links with a critical value can also change the nature of the network’s phase transition and prevent the network from suddenly collapsing, providing helpful guidance and novel insights into the design of more robust EINs.

The structure of this paper is as follows: Section 2 comprehensively overviews the proposed model. Section 3 details the analytical framework and presents the analytical solution. To verify the correctness of the theoretical analyses, we carry out extensive numerical simulations by configuring model-generated networks of artificial datasets, that is, ER-ER and SF-SF networks, as well as networks constructed using real datasets. The results of these simulations are presented in Section 4. Finally, Section 5 provides an in-depth discussion and analysis of the results obtained.

## 2. Model Description

**Figure 1:**
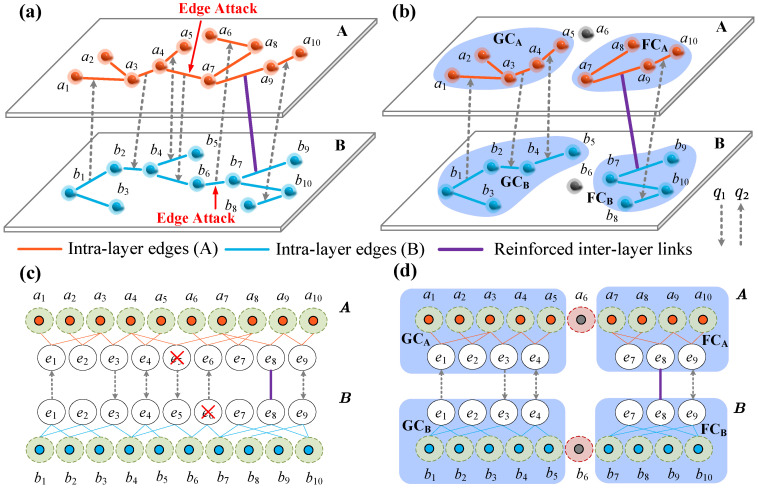
Schematic representation of an iterative process of a cascade of failures on an EIN with reinforced inter-layer links. The orange and blue solid lines within the subnets represent the intra-layer edges, the dashed lines with arrows between the subnets represent the inter-layer edges, and the purple line represents the reinforced inter-layer link. (**a**) The initial state, where an edge is randomly removed from network A and network B, i.e., edge $a_{4}-a_{7}$ and edge $b_{6}-b_{7}$ are initially failed, leading to the failure of edge $b_{4}-b_{6}$ and edge $a_{6}-a_{8}$ in their dependent networks, respectively. (**b**) The steady-state, where edges $a_{7}-a_{9}$ and $b_{7}-b_{9}$ can survive even if not in the GCs and support their surrounding nodes due to reinforced inter-layer links, and forming FCs. (**c**,**d**) show the bipartite networks representation of (**a**,**b**), where nodes and edges are indexed in the same way.

In this section, we illustrate the cascading failure process in the proposed two-layer EIN with reinforced inter-layer links, schematically shown in Figure 1. We initially consider two interdependent networks, denoted as A and B, with respective degree distributions $P_{A}\left(k_{A}\right)$ and $P_{B}\left(k_{B}\right)$. Network A comprises $N_{A}$ nodes and $E_{A}$ edges, while network B comprises $N_{B}$ nodes and $E_{B}$ edges. We also define that a fraction $q_{1}$ of edges in network A are randomly linked to edges in network B through inter-layer links, and the fraction $q_{2}$ in network B is similarly defined. Assume that each edge in each network has only one dependent edge. Especially if an edge in network A satisfies a one-to-one correspondence with an edge in network B, it is called a one-to-one no-feedback dependency relationship [4]. Due to the interdependency and interaction between the sub-networks, components from two subnets are associated through dependency links. In this work, a proportion of ρ among them is reinforced to improve the robustness of EINs. The edges supported by the reinforced inter-layer links remain functional and support the components they are in, even if they are not in the giant components (GCs). We define functional components (FCs) as smaller clusters containing at least one edge supported by a reinforced dependency link.

Figure 1a shows a schematic diagram of the EIN with reinforced inter-layer links in the initial state, where yellow and blue connecting edges are intra-layer edges, grey dashed lines with arrows are inter-layer links, and purple edges in bold are reinforced inter-layer links. The iterative cascading process of this model is initiated when the $1-p_{A}$ and $1-p_{B}$ edges in networks A and B are arbitrarily removed. After that, edges that suffer initial removal, failure of their dependent edges, or are not in the GC fail, and the failure propagates between the two networks until no more new edges fail. Figure 1b shows the final steady state reached after the network is attacked. In particular, an edge on either side of a reinforced dependency link can support its surrounding nodes and edges in its subnetwork to form FCs, e.g., $a_{7}-a_{9}$ and $b_{7}-b_{9}$ in Figure 1b, which are not in GCs but still survive, and the purple shaded portions of Figure 1b are the GCs and FCs surviving in network A and surviving GCs and FCs in network B. Furthermore, due to the partial dependencies within the networks, there are autonomous edges that do not have any dependent counterparts. These autonomous edges will remain intact if they are connected to the FC of their respective network. On the other hand, a regular edge survives if it and its corresponding dependent edges are connected to the giant component of their respective networks.

## 3. Mathematical Framework

In this section, we use self-consistent equations to solve the model based on generating functions. The generating functions of degree distributions and the corresponding residual degree distributions for the two layers are $G_{i0}\left(x\right)=\sum_{k_{i}}P_{i}\left(k_{i}\right)x^{k_{i}}$ and $G_{i1}\left(x\right)=\sum_{k_{i}}$$P_{i}\left(k_{i}\right)x^{k_{i}-1}/\langle k_{i}\rangle$, where i={A,B} and $\langle k_{i}\rangle$ represent the average degrees in networks A and B, respectively. We denote the sizes of the FC and GC of networks A and B as $P_{\infty }^{i}$ and $\mu_{\infty }^{i}$, respectively, which are also the probability that a randomly selected edge leads to the FC and GC. In addition, the auxiliary parameter *R* is defined as the probability of leading to the GC along a randomly chosen edge. For a single layer, a node with degree *k* and degree distribution P(k), if it does not belong to the GC, means that all its *k* edges are not connected to the GC, and the probability can be calculated as ${\left(1-R\right)}^{k}$. Then, average the probability over all possible degrees *k*; the probability that a node does not exist in the GC can be calculated as $1-\mu_{\infty }=\sum_{k=0}^{\infty }P\left(k\right){\left(1-R\right)}^{k}$, which can be further expressed using the generating functions as $\mu_{\infty }=1-G_{0}\left(1-R\right)$.

In EINs, the function of the networks is defined based on the edges, so it makes more sense to study this model based on bond percolation theory [10]. As an example, a fraction of 1−p of edges instead of a fraction of 1−p nodes is initially attacked in sub-networks. In network A, calculating the degree $k^{\prime }$ of a node in a damaged network requires removing $k^{\prime }$ edges from nodes in the original network with an initial degree of *k*, so we have $P\left(k^{\prime }\right)=\sum_{k=k^{\prime }}^{\infty }P\left(k\right)\left(\begin{array}{c} k\\ k^{\prime } \end{array}\right)P\left(k^{\prime }\right){\left(1-p\right)}^{k-k^{\prime }}$. Therefore, the degree distribution becomes $\sum_{k^{\prime }=0}^{\infty }P\left(k^{\prime }\right)R^{k^{\prime }}$, and the residual degree distribution becomes $\sum_{k^{\prime }=1}^{\infty }\frac{P\left(k^{\prime }\right)k^{\prime }}{p\langle k\rangle }R^{k^{\prime }-1}$. Using the generating function, we can represent the auxiliary parameter *R* in the network after an initial attack as follows:

(1) $$R=\sum_{k^{\prime }}\frac{P\left(k^{\prime }\right)k^{\prime }}{\left.\langle k_{A}^{\prime }\right.\rangle }\left[1-{\left(1-R\right)}^{k^{\prime }-1}\right]=p\cdot \left[1-G_{1}\left(1-R\right)\right].$$

Following the single-layer network method, we extend the theory to two-layer EINs with reinforced inter-layer links. We first define two auxiliary parameters, $R_{A}$ and $R_{B}$, which denote the probability that any edge in network A and network B leads to an FC, respectively. We further assume that a proportion of $q_{1}$ edges in network A depend on the edges in network B, i.e., the remaining $1-q_{1}$ proportional edges are autonomous, and a proportion of $q_{2}$ edges in network B depend on the edges in network A, i.e., the remaining $1-q_{2}$ proportional edges are autonomous. Firstly, a fraction of $1-p_{A}$ edges in network A and a fraction of $1-p_{B}$ edges in network B are removed to trigger a cascading process. For simplification, we assume $p_{A}=p_{B}=p$. Thus, any edge in network A leading to an FC in the network is as follows: ($E_{1}$) edge *l* has no dependent edges. It is connected to an FC in the network. ($E_{2}$) edge *l* has a dependent edge $l^{\prime }$. The dependent edges between the two are not reinforced, but both edge *l* and edge $l^{\prime }$ are connected to an FC in the respective network. ($E_{3}$) edge *l* and the dependent edge between edges $l^{\prime }$ are reinforced. The probability of ($E_{1}$) can be calculated as follows:

(2) $$P\left(E_{1}\right)=p\cdot \left(1-q_{1}\right)\cdot \sum_{k^{\prime }}\frac{P_{A}\left(k\right)k}{\left.\langle k_{A}\right.\rangle }\left[1-{\left(1-R_{A}\right)}^{k-1}\right],$$

where $P_{A}\left(k\right)k/\langle k\rangle$ represents the probability of the node *u* with the degree of *k*, which can be further expressed by generating functions as

(3) $$P\left(E_{1}\right)=p\cdot \left(1-q_{1}\right)\cdot \left[1-G\left(1-R_{A}\right)\right],$$

Next, we calculate the probability of event $\left(E_{2}\right)$. It is important to note that if we randomly select an edge *l* in network A that leads to the FC in EINs, we must also ensure that the corresponding edge $l^{\prime }$ in network B is also part of the FC. Consequently, the central issue is to determine the probability that a randomly selected edge in network B belongs to the FC. This probability can be readily deduced from the complementary probability that neither endpoint of an edge in network B is part of the FC. Based on the preceding analysis, the probability that an edge is not part of the FC can be derived as

(4) $$\begin{array}{cc} P(E_{2}) & =p\cdot q_{1}\left(1-\rho \right)\cdot \sum_{k}\frac{P_{A}\left(k\right)k}{\langle k\rangle }\left[1-{\left(1-R_{A}\right)}^{k-1}\right]\cdot p\cdot \left\{1-{\left[\sum_{k^{\prime }}\frac{P_{B}\left(k^{\prime }\right)k^{\prime }}{\left.\langle k_{B}^{\prime }\right.\rangle }{\left(1-R_{B}\right)}^{k^{\prime }-1}\right]}^{2}\right\}\\ \; & =p^{2}\cdot q_{1}\left(1-\rho \right)\cdot \left[1-G_{1}\left(1-R_{A}\right)\right]\left[1-G_{1}{\left(1-R_{B}\right)}^{2}\right], \end{array}$$

where 1−ρ means that the probability that the selected pair of edges *l* and $l^{\prime }$ has not been strengthened by the reinforced dependency link, ${\left[\sum_{k^{\prime }}\frac{P_{B}(k^{\prime })k^{\prime }}{\left.\langle k_{B}^{\prime }\right.\rangle }{\left(1-R_{B}\right)}^{k^{\prime }-1}\right]}^{2}$ means that the probability of both ends of edge $l^{\prime }$ in network B are not in the FCs.

The probability that the selected pair of edges *l* and $l^{\prime }$ are strengthened by the reinforced dependency link and lead to the FC can be denoted as follows:

(5) $$P\left(E_{3}\right)=p^{2}q_{1}\rho ,$$

We can represent the expression of $R_{A}$ by using the addition rule; thus, we have $R_{A}=P\left(E_{1}\right)+P\left(E_{2}\right)+P\left(E_{3}\right)$ as

(6) $$R_{A}=p\left\{pq_{1}\rho +\left[1-G_{A1}\left(1-R_{A}\right)\right]\left\{\left(1-q_{1}\right)+p\cdot q_{1}\left(1-\rho \right)\cdot \left[1-G_{B1}{\left(1-R_{B}\right)}^{2}\right]\right\}\right\},$$

And by the same token, a similar calculation procedure of $R_{B}$ can be derived as $R_{A}$, as follows:

(7) $$R_{B}=p\left\{pq_{2}\rho +\left[1-G_{B1}\left(1-R_{B}\right)\right]\left\{\left(1-q_{2}\right)+p\cdot q_{2}\left(1-\rho \right)\cdot \left[1-G_{A1}{\left(1-R_{A}\right)}^{2}\right]\right\}\right\},$$

Since it is the edges that are deleted in the network and there is no dependency between the nodes of the two networks, the order parameter $P_{\infty }^{A}\left(P_{\infty }^{B}\right)$ is not directly affected, so the size of the FCs in the network can be calculated as

(8) $$\left\{\begin{array}{c} P_{\infty }^{A}=1-G_{A0}\left(1-R_{A}\right)\\ P_{\infty }^{B}=1-G_{A0}\left(1-R_{B}\right) \end{array}\right.,$$

For comparison, we also define the probability of a randomly selected edge leading to the GC in network A as ${\tilde{R}}_{A}$, and the probability of a randomly selected edge leading to the GC in network B as ${\tilde{R}}_{B}$. The expressions of ${\tilde{R}}_{A}$ and ${\tilde{R}}_{B}$ are shown as Equation (9), which can be calculated with the values of $R_{A}$ and $R_{B}$ obtained by solving Equations (6) and (7) as

(9) $$\left\{\begin{array}{c} {\tilde{R}}_{A}=p\left[1-G_{A1}\left(1-{\tilde{R}}_{A}\right)\right]\left\{1-q+p\left\{q\rho +q\left(1-\rho \right)\cdot \left[1-G_{B1}{\left(1-R_{B}\right)}^{2}\right]\right\}\right\}\\ {\tilde{R}}_{B}=p\left[1-G_{B1}\left(1-{\tilde{R}}_{B}\right)\right]\left\{1-q+p\left\{q\rho +q\left(1-\rho \right)\cdot \left[1-G_{A1}{\left(1-R_{A}\right)}^{2}\right]\right\}\right\}. \end{array}\right.$$

After that, the size of the GCs, i.e., $\mu_{\infty }^{A}$ and $\mu_{\infty }^{B}$, of networks A and B can be calculated as

(10) $$\left\{\begin{array}{c} \mu_{\infty }^{A}=1-G_{A0}\left(1-{\tilde{R}}_{A}\right)\\ \mu_{\infty }^{B}=1-G_{A0}\left(1-{\tilde{R}}_{B}\right) \end{array}\right..$$

Then, by combining Equations (6), (7), and (9), we can obtain the numerical solutions of ${\tilde{R}}_{A}$ and ${\tilde{R}}_{B}$ of networks A and B; moreover, we can obtain the values of $\mu_{A}$ and $\mu_{B}$ by substituting the values of $R_{A}$ and $R_{B}$ in Equation (10).

For the discontinuous transition, the expressions of Equations (6) and (7) can be rewritten as $R_{A}^{I}=F_{1}\left(p_{c}^{I},R_{B}^{I},\rho \right)$ and $R_{B}^{I}=F_{1}\left(p_{c}^{I},R_{A}^{I},\rho \right)$, which also satisfies the condition at $p_{c}^{I}$, as follows:

(11) $$\frac{\partial F_{1}\left(p_{c}^{I},R_{B}^{I},\rho \right)}{\partial R_{B}^{I}}\cdot \frac{\partial F_{2}\left(p_{c}^{I},R_{A}^{I},\rho \right)}{\partial R_{A}^{I}}=1,$$

where the $R_{A}$ and $R_{B}$ curves are tangent to each other in the first quadrant of the $R_{A}-R_{B}$ plane at $\left(R_{A}^{I},R_{B}^{I}\right)$.

For the continuous transition, we consider the condition where the size of GC decreases continuously as the proportion of removed nodes increases at the critical transition point $p_{c}^{\mathrm{II}}$; at the same time, the GC disappears. Then, we have ${\tilde{R}}_{A}\to 0$ and ${\tilde{R}}_{B}\to 0$. We first perform a Taylor expansion of Equation (6) at ${\tilde{R}}_{A}\to 0$, as follows:

(12) $$R_{A}=H_{1}^{\prime }\left(p_{c}^{I},R_{B},{\tilde{R}}_{A}\right)\tilde{x}+\frac{1}{2}H_{1}^{\prime \prime }\left(p_{c}^{I},R_{B},{\tilde{R}}_{A}\right){\tilde{R}}_{A}^{2}+o\left({\tilde{R}}_{A}^{3}\right),$$

which leads to

(13) $$H_{1}^{\prime }\left(p_{c}^{\mathrm{II}},R_{B},0\right)=p_{c}^{\mathrm{II}}\left(1-q\right)+{\left(p_{c}^{\mathrm{II}}\right)}^{2}\left\{q\rho +q\left(1-\rho \right)\left[1-G_{B0}\left(1-R_{B}\right)\right]\right\}=\frac{1}{\;\;G_{A1}^{\prime }\left(1\right)},$$

By combining Equations (6), (7), and (13), we can obtain the numerical solutions of $p_{c}^{\mathrm{II}}$. In particular, we discuss the following cases, namely q=1, ρ=0, and q=0. For the first case, the proposed model is simplified to the model of the original EIN, as presented in ref. [19], where there is no solution for $p_{c}^{\mathrm{II}}$, indicating that there is no continuous phase transition. For another case, q=0 means that there are no interdependence relations between networks; the proposed model is equivalent to the single-layer model, where the $p_{c}^{\mathrm{II}}$ can be solved by applying $p_{c}^{\mathrm{II}}=1/G_{1}^{\prime }\left(1\right)$, which corresponds to the previous study [4].

For simplification, we assume in the symmetric case that $p_{A}=p_{B}=p$, $q_{A}=q_{B}=q$, $\langle k_{A}\rangle =\langle k_{B}\rangle =\langle k\rangle$. Based on the above assumptions, we have $R_{A}=R_{B}=R\equiv F\left(p,R\right)$, $P_{\infty }^{A}=P_{\infty }^{B}=P_{\infty }$ as Equation (14) for the calculation of FCs and we have ${\tilde{R}}_{A}={\tilde{R}}_{B}=\tilde{R}\equiv F\left(p,\tilde{R}\right)$, $\mu_{\infty }^{A}=\mu_{\infty }^{B}=\mu_{\infty }$ as Equation (14) for the calculation of GCs.

(14) $$\left\{\begin{array}{c} R=g\left(R\right)+h\left(R\right)p^{2}\equiv F\left(p,R\right)\\ P_{\infty }=1-G_{0}\left(1-R\right) \end{array}\right.,$$

where $g\left(R\right)=\left(1-q\right)\left[1-G_{1}\left(1-R\right)\right]$, $h\left(R\right)=q\rho +q\left(1-\rho \right)\cdot \left[1-G_{1}\left(1-R\right)\right]\left[1-G_{1}{\left(1-R\right)}^{2}\right]$.

We have $\partial F\left(p^{I},R^{I}\right)/\partial R^{I}=1$ at the onset of the discontinuous transitions (i.e., $p=p_{c}^{I}$), namely

(15) $$h^{\prime }\left(R^{I}\right){\left(p_{c}^{I}\right)}^{2}+g^{\prime }\left(R^{I}\right)p_{c}^{I}-1=0,$$

where $G_{1}^{\prime }\left(1-R^{I}\right)$ is the derivative of $G_{1}\left(1-R\right)$ that is taken with respect to $1-R^{I}$ as a whole, which can be be further deduced to $g^{\prime }\left(R^{I}\right)$ and $h^{\prime }\left(R^{I}\right)$ as $g^{\prime }\left(R^{I}\right)=\left(1-q\right)G_{1}^{\prime }\left(1-R^{I}\right)$ and $h^{\prime }\left(R^{I}\right)=q\left(1-\rho \right)\cdot \left\{G_{1}^{\prime }\left(1-R^{I}\right)\left[1-G_{1}{\left(1-R^{I}\right)}^{2}\right]+2G_{1}\left(1-R^{I}\right)G_{1}^{\prime }\left(1-R^{I}\right)\right\}$.

Solving $p_{c}^{I}$ from Equation (15), we have

(16) $$p_{c}^{I}\left(R^{I}\right)=\frac{-g^{\prime }\left(R^{I}\right)+\sqrt{{\left(g^{\prime }\left(R^{I}\right)\right)}^{2}+4h^{\prime }\left(R^{I}\right)}}{2h^{\prime }\left(R^{I}\right)}.$$

It is worth noting that $R^{I}$ in Equation (16) should also satisfy Equation (14) at $p_{c}^{I}$ simultaneously, as follows:

(17) $$R^{I}=g\left(R^{I}\right)p_{c}^{I}+h\left(R^{I}\right){\left(p_{c}^{I}\right)}^{2},$$

Equations (16) and (17) can be utilized to solve for $p_{c}^{I}$ and $R^{I}$. Then, consider the solution of critical $\rho^{*}$, where the critical phase transition threshold $p_{c}^{I}=p_{c}^{\mathrm{II}}$. Deriving both sides of Equation (17), the equation still holds at $R^{I}$ as

(18) $$1=g^{\prime }\left(R^{I}\right)p_{c}^{I}+h^{\prime }\left(R^{I}\right){\left(p_{c}^{I}\right)}^{2}+\left[g\left(R^{I}\right)+2h\left(R^{I}\right)p_{c}^{I}\right]\frac{dp_{c}^{I}}{dR^{I}},$$

which can be further simplified by substituting $h^{\prime }\left(R^{I}\right){\left(p_{c}^{I}\right)}^{2}+g^{\prime }\left(R^{I}\right)p_{c}^{I}=1$ as in Equation (15), which is described as follows:

(19) $$0=\left[g\left(R^{I}\right)+2h\left(R^{I}\right)p_{c}^{I}\right]\frac{dp_{c}^{I}}{dR^{I}},$$

For any ρ>0, our definitions of g(x) and h(x) show that they are positive for any x∈[0,1], and hence $g\left(R^{I}\right)+2h\left(R^{I}\right)p_{c}^{I}$ is also positive, so the condition for the above equation to hold is

(20) $$\frac{dp_{c}^{I}}{dR^{I}}=0.$$

Using $p_{c}^{I}$ obtained from Equation (16), we can further transform the determinant into the following form:

(21) $$g^{\prime \prime }\left(R^{I}\right)h^{\prime }\left(R^{I}\right)\left[g^{\prime }\left(R^{I}\right)-\sqrt{△}\right]+\left[g^{\prime }\left(R^{I}\right)\sqrt{△}+2h^{\prime }\left(R^{I}\right)-△\right]h^{\prime \prime }\left(R^{I}\right)=0,$$

where $△={\left(g^{\prime }\left(R^{I}\right)\right)}^{2}+4h^{\prime }\left(R^{I}\right)$.

And when $\rho >\rho^{*}$, there is no solution for $R^{I}$. Moreover, the network disintegrates in the form of a continuous phase transition. Therefore, we have $\tilde{R}\to 0$ at $p\to p_{c}^{\mathrm{II}}$. Thus, Equation (13) can be expressed as

(22) $$H^{\prime }\left(p_{c}^{\mathrm{II}},R^{\mathrm{II}},0\right)=p_{c}^{\mathrm{II}}\left(1-q\right)+{\left(p_{c}^{\mathrm{II}}\right)}^{2}\left\{q\rho +q\left(1-\rho \right)\cdot \left[1-G_{1}{\left(1-R^{\mathrm{II}}\right)}^{2}\right]\right\},$$

which can be concisely rewritten in the following form:

(23) $$H^{\prime }\left(p_{c}^{\mathrm{II}},R^{\mathrm{II}},0\right)=C_{1}p_{c}^{\mathrm{II}}+f\left(R^{\mathrm{II}}\right){\left(p_{c}^{\mathrm{II}}\right)}^{2}=\frac{1}{G_{1}^{\prime }\left(1\right)},$$

where $C_{1}=1-q$, $f\left(R^{\mathrm{II}}\right)=q\rho +q\left(1-\rho \right)\cdot \left[1-G_{1}{\left(1-R^{\mathrm{II}}\right)}^{2}\right]$. Then, we can obtain the solutions of $p_{c}^{\mathrm{II}}$ and $R^{\mathrm{II}}$ as

(24) $$\left\{\begin{array}{c} p_{c}^{\mathrm{II}}\left(R^{\mathrm{II}}\right)=\frac{-C_{1}+\sqrt{C_{1}^{2}{G_{1}}^{\prime }\left(1\right)+4f\left(R^{\mathrm{II}}\right)}}{2f\left(R^{\mathrm{II}}\right)\sqrt{{G_{1}}^{\prime }\left(1\right)}}\\ R^{\mathrm{II}}=g\left(R^{\mathrm{II}}\right)p_{c}^{\mathrm{II}}+h\left(R^{\mathrm{II}}\right){\left(p_{c}^{\mathrm{II}}\right)}^{2}. \end{array}\right.$$

After that, the critical threshold of $\rho^{*}$ can be calculated by combing Equations (16) and (24).

## 4. Results

In this section, we illustrate theoretical solution and simulation results on two synthetic datasets, that is, Erdős–Rényi (ER) and scale-free (SF) networks, and an example of a real-world system to verify the analysis proposed above. The parameters of the EIN are set as $N_{A}=N_{B}=10^{6}$ for the number of nodes in layers A and B, with $E_{A}=E_{B}$ ensuring equal numbers of edges in both layers. In the ER-ER symmetric EIN, the degree distributions of networks A and B satisfy $P_{A}\left(k_{A}\right)=e^{-\langle k_{A}\rangle }{\langle k_{A}\rangle }^{k_{A}}/k_{A}!$ and $P_{B}\left(k_{B}\right)=e^{-\langle k_{B}\rangle }{\langle k_{B}\rangle }^{k_{B}}/k_{B}!$, and the corresponding generating functions are $G_{A0}\left(x\right)=G_{A1}\left(x\right)=e^{\langle k_{A}\rangle \left(x-1\right)}$, $G_{B0}\left(x\right)=G_{B1}\left(x\right)=e^{\langle k_{B}\rangle \left(y-1\right)}$. In the SF-SF symmetric EIN, the degree distribution satisfies $P\left(k\right)=ck^{-\lambda }$ for both networks, where $k_{\min}\leq k\leq K$, and λ is the distribution’s degree exponent. As $P\left(k\right)={\left(k_{\min}/k\right)}^{\lambda -1}-{\left(k_{\min}/k+1\right)}^{\lambda -1}$ is a reasonable approximation for the degree distribution, the generating functions of SF networks can be calculated as [38]

(25) $$\left\{\begin{array}{c} G_{0}\left(x\right)=\sum_{k_{\min}}^{K}\left[{\left(\frac{k_{\min}}{k}\right)}^{\lambda -1}-{\left(\frac{k_{\min}}{k+1}\right)}^{\lambda -1}\right]\cdot x^{k}\\ G_{1}\left(x\right)=\sum_{k_{\min}}^{K}k\cdot \left[{\left(\frac{k_{\min}}{k}\right)}^{\lambda -1}-{\left(\frac{k_{\min}}{k+1}\right)}^{\lambda -1}\right]\cdot x^{k-1}/\langle k\rangle \end{array}\right.$$

For simplification, we assume that $p_{A}=p_{B}=p$, $q_{A}=q_{B}=q$, $\langle k_{A}\rangle =\langle k_{B}\rangle =\langle k\rangle$.

Based on the theoretical analysis above, by combining Equations (6)–(10), we can calculate the magnitude of the FCs ($P_{\infty }$) and the magnitude of the GCs ($\mu_{\infty }$) under ER-ER and SF-SF networks. For discontinuous phase transitions, Equations (6) and (7) meet tangentially, as shown in Equation (10) at the phase transition point $p_{c}^{I}$. For continuous phase transitions, the phase transition point $p_{c}^{\mathrm{II}}$ can be solved by combining Equations (24) and (25). From the above analysis, we learn that there is a critical value of $\rho^{*}$, which can change the phase transition type of the system. The critical $\rho^{*}$ can be determined by assuming $p_{c}^{I}=p_{c}^{\mathrm{II}}$, i.e., combining Equations (16), (17), and (24).

**Figure 2:**
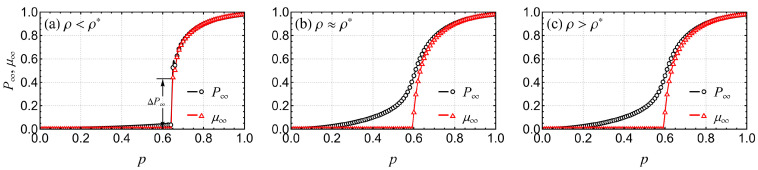
Percolation characteristics for symmetric fully interdependent ER-ER EINs with reinforced inter-layer links with $\langle k\rangle =4$, q=1, and (**a**) $\rho =0.02<\rho^{*}$; (**b**) $\rho =0.08\approx \rho^{*}$; or (**c**) $\rho =0.15>\rho^{*}$. Solid lines are theoretical solutions that were obtained by combining Equations (6)–(10), and symbols are simulation results from a network with $N_{A}=N_{B}=10^{6}$ nodes. Note that, when $\rho \leq \rho^{*}$, both $P_{\infty }$ and $\mu_{\infty }$ undergo a discontinuous phase transition at the same $p_{c}^{I}$. However, when $\rho >\rho^{*}$, $\mu_{\infty }$ undergoes a continuous transition, and $P_{\infty }$ is continuous and free of phase transitions due to the presence of the FCs, except the GC.

In addition to solving the special value $\rho^{*}$ numerically, we can also understand its meaning through the percolation properties of interdependent systems. Figure 2 gives the sizes of FCs ($P_{\infty }$) and GCs ($\mu_{\infty }$), which vary as a function of *p* for different values of ρ. When $\rho <\rho^{*}$ (Figure 2a), $P_{\infty }$ and $\mu_{\infty }$ undergo a discontinuous phase transition at the same $p_{c}^{I}$, and there is a jump of $△P_{\infty }$ at the same phase transition point $p_{c}^{I}$ (marked with arrows in Figure 2a). As ρ increases towards $\rho^{*}$, it reduces the jump size $△P_{\infty }$ of the FCs and reduces the percolation threshold $p_{I}^{c}$ at which the jump occurs. When $\rho =\rho^{*}$ (Figure 2b), the phase transition type of the $\mu_{\infty }$ changes from discontinuous to continuous, while $P_{\infty }$ is almost a continuous transition at this point. Finally, if $\rho >\rho^{*}$ (Figure 2c), $\mu_{\infty }$ undergoes a continuous phase transition, which is unobservable from the point of view of $P_{\infty }$; therefore, we call this behavior of $P_{\infty }$ a transition-free type. Note that $\rho^{*}$ takes its maximum value $\rho_{max}^{*}$ at q=1. Furthermore, $\rho =\rho^{*}$ essentially defines the boundary between the discontinuous transition type and transition-free type of order parameter $P_{\infty }$, as well as the boundary between the discontinuous transition type and continuous type of order parameter $\mu_{\infty }$.

**Figure 3:**
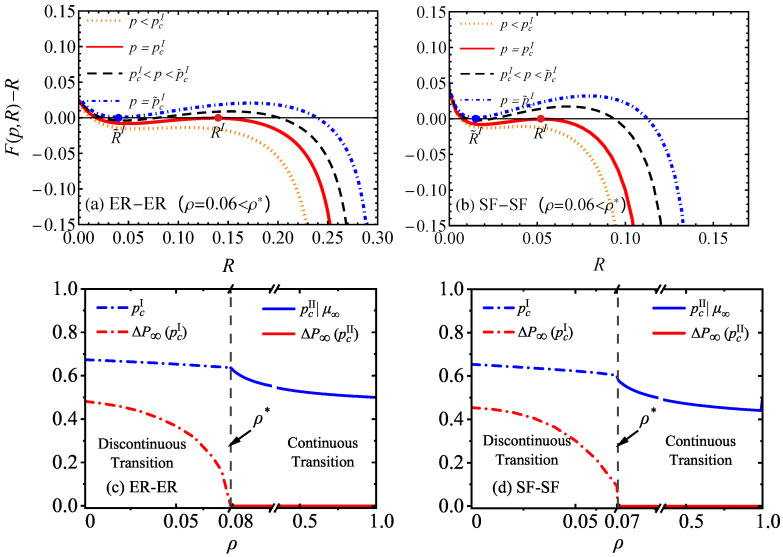
Graphical solutions of critical point and the phase transitions properties for the symmetric ER-ER [$p\left(k\right)=e^{-\langle k\rangle }{\langle k\rangle }^{k}/k!$, $\langle k\rangle =4$] and SF-SF [$p\left(k\right)∼k^{-\gamma }$, λ=2.7, $k_{min}=2$, K=2000] EINs with reinforced inter-layer links. F(p,R)−R with varying values of *p* with fixed $\rho =0.06<\rho^{*}$ and q=1 in the case of (**a**) the ER-ER EINs and (**b**) SF-SF EINs, with varying values of *p*. The discontinuous transition threshold $p_{c}^{I}$ (indicated by the blue dot-dashed line), continuous transition threshold $p_{c}^{\mathrm{II}}$ (indicated by the blue solid line), and $△P_{\infty }$ (indicated by the red lines) are plotted as functions of ρ for (**c**) ER-ER EINs and (**d**) SF-SF EINs.

Combining Equations (16) and (17), we can obtain the solutions of $R^{I}$ and $p_{c}^{I}$. As shown in Figure 3a,b, there exist two solutions, $R^{I}$ and ${\tilde{R}}^{I}$, corresponding to $p_{c}^{I}$ and ${\tilde{p}}_{c}^{I}$, respectively. Here, we choose the first occurrence of the non-trivial solution $R^{I}$ and its corresponding $p_{c}^{I}$ as the equation solution. If $\rho =\rho^{*}$, there is a unique solution for $R^{I}$. Next, the thresholds $p_{c}^{I}$ for discontinuous phase transitions and $p_{c}^{\mathrm{II}}$ for continuous phase transitions of the network GCs ($\mu_{\infty }$) and the jumps in the sizes of the FCs $△P_{\infty }$ as the proportion of reinforced inter-layer links ρ are represented in Figure 3c,d. Furthermore, since the analytical solution of $P_{\infty }$ is too complex and difficult to derive, it is derived here from the difference of neighboring $P_{\infty }$ near the phase transition point, with the step size set to 0.001. It can be seen that $△P_{\infty }$ reduces to 0 at $\rho^{*}$, further proving that $\rho^{*}$ are the boundaries that alter the phase transition types of FCs from discontinuous to transition-free types, and alters the phase transition types of GCs from discontinuous to continuous ones, which is also the minimum ratio of reinforced inter-layer links required to prevent catastrophic network collapse.

**Figure 4:**
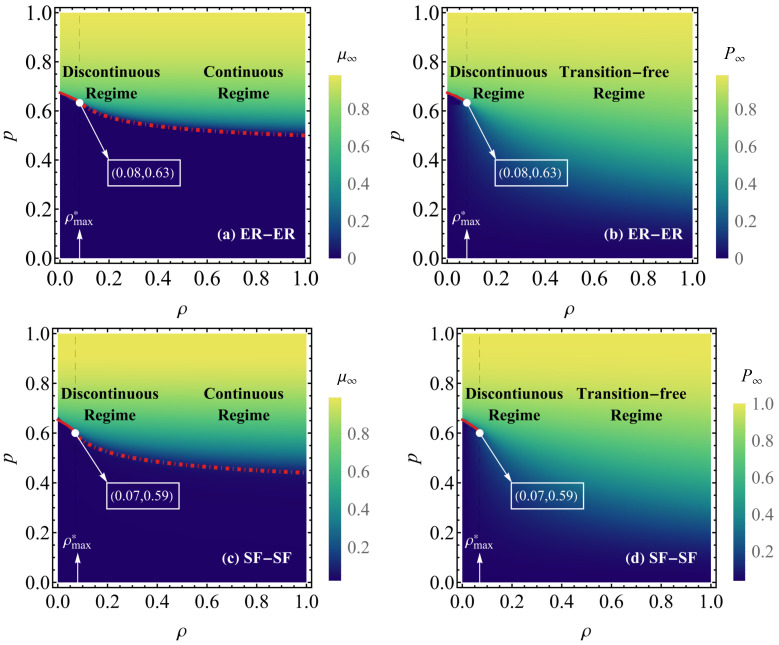
Phase diagrams in plane (ρ,p) in symmetric ER-ER (**a**,**b**) and SF-SF (**c**,**d**) EINs with reinforced inter-layer links. Panels (**a**,**c**) illustrate the sizes of $\mu_{\infty }$ within networks, which are divided by a gray dashed line into two regimes, that is, discontinuous and continuous regimes, and the red solid line left to the boundary represent the discontinuous threshold $p_{c}^{I}$. The red dash-dotted line right to the boundary represents the continuous threshold $p_{c}^{\mathrm{II}}$. Panels (**b**,**d**) illustrate the sizes of $P_{\infty }$ within networks, which are divided by a black dashed line into two regimes, that is, discontinuous and transition-free regimes, and the red solid line left to the boundary represents the discontinuous threshold $p_{c}^{I}$, while when $\rho >\rho^{*}$, there is no phase transition for FCs. The network parameters are set as $N_{A}=N_{B}=10^{6}$, $\langle k\rangle =4$, and q=1.

Next, Figure 4 systematically examines the sizes of $P_{\infty }$ and $\mu_{\infty }$ in networks A and B in the plane (p,γ) of ER-ER (Figure 4a,b) and SF-SF (Figure 4c,d) EINs with reinforced inter-layer links when q=1. In Figure 4a,c, it can be seen that as ρ increases, the GC size of the network ($\mu_{\infty }$) gradually increases with the same fraction of reserved nodes *p*; the whole plane is divided by a red line into two distinct regimes, with the functional regime above the red line and the collapsed regime below the red line. At the critical value $\rho^{*}$, there is a black dashed line as a boundary dividing the figure into discontinuous and continuous transition regimes, where the red solid line to the left of the black dashed line is the value of $p_{c}^{I}$ for the discontinuous transition of the network. The red dashed line to the right of the black dashed line is the value of $p_{c}^{\mathrm{II}}$ for the continuous transition of the network. In Figure 4b,d, it can be seen that the FC size $P_{\infty }$ of the network gradually increases as the value of ρ increases, with the same fraction of reserved nodes *p*. There exists a black dashed line as a boundary dividing the figure into the discontinuous transition and the transition-free regimes at a critical value of $\rho^{*}$, where the red solid line to the left of the black dashed line is the value of $p_{c}^{I}$ for the discontinuous transition of the network. As *p* decreases, for $\rho \leq \rho^{*}$, $\mu_{\infty }$ remains positive for *p* values greater than $p_{c}^{I}$ and continuously decreases to 0 as *p* approaches $p_{c}^{I}$. On the other hand, when $\rho >\rho^{*}$, $\mu_{\infty }$ remains positive for *p* values greater than $p_{c}^{\mathrm{II}}$ and discontinuously decreases to 0 as *p* approaches $p_{c}^{\mathrm{II}}$. For comparison, $P_{\infty }$ remains positive for *p* greater than $p_{c}^{I}$ and decreases discontinuously to zero as *p* approaches $p_{c}^{I}$. On the other hand, $P_{\infty }$ remains positive for *p* values greater than $p_{c}^{\mathrm{II}}$ as $\rho >\rho^{*}$ and decreases to a certain value as *p* approaches $p_{c}^{\mathrm{II}}$.

**Figure 5:**
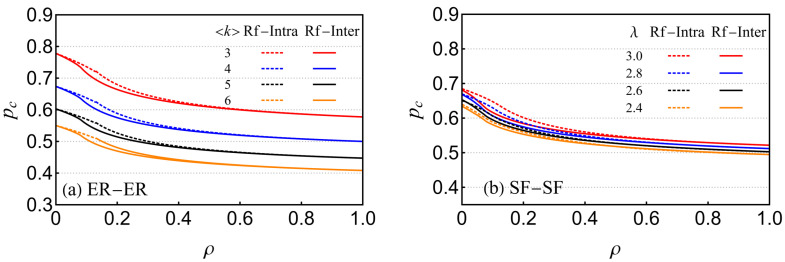
The phase diagram of ER-ER EINs and SF-SF EINs with reinforced links under (**a**) different values of $\langle k\rangle$ for ER-ER EINs and (**b**) different values of λ for SF-SF EINs versus ρ with q=1, where different reinforcement strategies are applied, that is, intra-layer reinforced (dashed lines) and inter-layer reinforced (solid lines).

Figure 5a illustrates the critical percolation transition threshold of the ER-ER EIN with reinforced links, including reinforced inter-layer links and intra-layer links, for different average degrees. The closed region between the $p_{c}$ curve and the axes indicates the collapse state where the network is destroyed. We can observe that this region decreases as the average number of neighbors (nodes and edges) per node in the ER-ER network increases, implying that the higher the $\langle k\rangle$, the more robust the EIN. As shown in Figure 5b, we performed a similar experiment on the SF-SF network, varying the value of λ to observe its behavior. The results show that the collapsed area expands as the value of λ increases, indicating that increasing λ has a negative impact on improving the robustness of the network. In addition, Figure 5a,b show that increasing the proportion of ρ leads to a decrease in the percolation thresholds under different values of $\langle k\rangle$ and λ, meaning that the increase in proportional reinforced inter-layer links can effectively protect the network nodes and edges from sudden catastrophic collapses. The results also showed that in Figure 5, compared with reinforced intra-layer links, reinforced inter-layer links have a greater reinforced efficiency.

**Figure 6:**
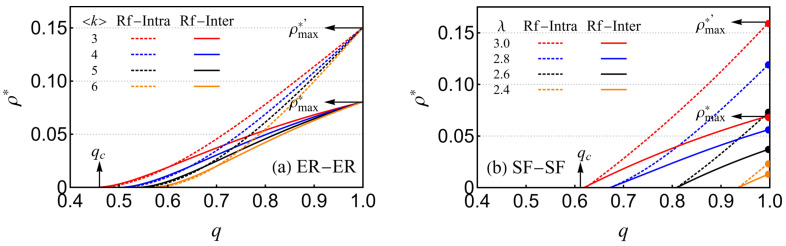
Relationship between the critical proportion of reinforced links $\rho^{*}$ and the coupling strength *q*. (**a**) required $\rho^{*}$ versus *q* in the case of ER-ER EINs with different values of the average degree $\langle k\rangle$. (**b**) Required $\rho^{*}$ versus *q* in the case of SF-SF EINs with different values of the degree exponent λ, where different reinforcement strategies are applied, that is, intra-layer reinforced (dashed lines) and inter-layer reinforced (solid lines).

We then analyzed the relationship between parameter *q* and critical threshold $\rho^{*}$, varying *q* and imposing the condition $p_{c}^{I}=p_{c}^{\mathrm{II}}$. This approach enabled us to construct the plot of $\rho^{*}$ as a function of *q*, as depicted in Figure 6. As illustrated in Figure 6a for the ER-ER network, an increase in *q* corresponds to an increase in $\rho^{*}$. Specifically, $\rho^{*}$ attains its maximum value, denoted as $\rho_{\max}^{*}$, when q=1. This observation is attributed to the negative effect of increased coupling strength on the network’s robustness. When the proportion ρ of reinforced inter-layer links is held constant, a smaller average degree 〈k〉 corresponds to a lower value of *q*. Notably, regardless of the specific 〈k〉, all curves converge to a common maximum $\rho_{\max}^{*}$ and $\rho_{\max}^{*\prime }$ of approximately 0.808 for the inter-layer reinforcement strategy and about 0.164 for the intra-layer reinforcement strategy, both at q=1. In the SF-SF network, as shown in Figure 6b, the maximum values of $\rho^{*}$ and $\rho_{\max}^{*\prime }$ vary with different values of the exponent λ. The value of $q=q_{c}$ at the intersection with the ρ axis represents the critical coupling strength that prevents the catastrophic collapse of the network. At this point, the model is effectively equivalent to a conventional one without the introduction of reinforced inter-layer links (ρ=0). Comparatively, the lower values of $\rho_{\max}^{*}$ for a given *q* indicate that reinforcing inter-layer links is more effective than reinforcing intra-layer links.

**Figure 7:**
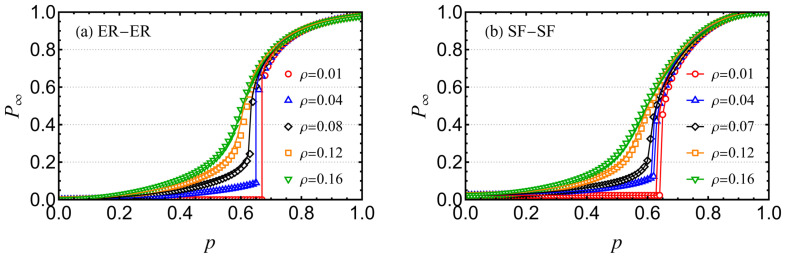
Cascading failures of symmetrical (**a**) ER-ER EINs and (**b**) SF-SF EINs with reinforced inter-layer links under different values of ρ, where the networks are composed of artificial datasets. The solid lines and symbols are simulated results and the corresponding theoretical predictions. The other network parameters are set as $N_{A}=N_{B}=10^{6}$, $\langle k_{A}\rangle =\langle k_{B}\rangle =4$, and $\lambda_{A}=\lambda_{B}=2.7$.

Furthermore, in this study, we utilize both synthetic and realistic network datasets for theoretical analysis and experimental validation. Figure 7a,b depict cascading failure scenarios on symmetric ER-ER and SF-SF EINs with reinforced inter-layer links. In these simulations, the average degree of the ER networks is set to $\langle k_{A}\rangle =\langle k_{B}\rangle =4$, and the scaling parameter of the SF networks is $\lambda_{A}=\lambda_{B}=2.7$. The results show that $P_{\infty }$ increases with the fraction of reserved edges *p*, following an initial attack when ρ is held constant. Conversely, for a fixed *p*, $P_{\infty }$ increases as ρ rises, transitioning to a phase without abrupt collapse once ρ surpasses a critical value $\rho^{*}$. Concurrently, the network’s phase transition behavior changes to a continuous transition, a phenomenon not depicted here but described in Figure 4. Symbolic lines represent simulation results, whereas solid lines represent theoretical predictions, demonstrating a good match. These findings confirm that increasing the proportion of reinforced inter-layer links enhances the robustness of the EIN. This outcome aligns with the conclusion drawn for non-interacting networks (NINs), where a specific proportion of reinforced nodes is crucial for mitigating the risk of sudden network collapse. Specifically, for q=1, the proportion of reinforced inter-layer links required to prevent abrupt collapse is greater than $\rho_{\max}^{*}\approx 0.0808$ in ER-ER networks and approximately 0.0764 in SF-SF networks.

**Figure 8:**
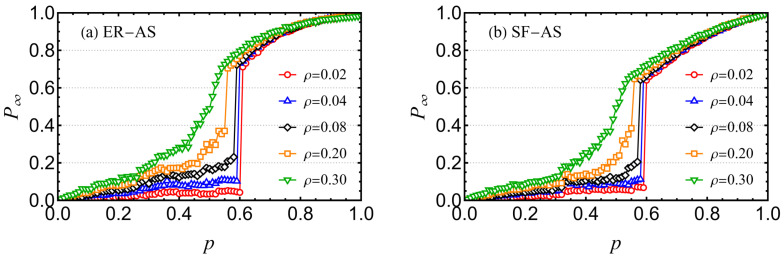
Cascading failures of (**a**) ER-AS EINs and (**b**) SF-AS EINs with reinforced inter-layer links under different values of ρ, where the networks are composed of artificial and real-world datasets. The symbols represent simulation results. The other network parameters are set as $N_{A}=N_{B}=6474$, $E_{A}=E_{B}=13,895$.

The proposed mathematical framework was evaluated using a real-world AS routing network dataset obtained from the University of Oregon’s Route Views Project [39]. Due to the scarcity of detailed information about the topologies and interdependencies of the networks interacting with the AS network, we adopted a qualitative approach. Specifically, we interconnected the AS router network with ER and SF networks of equivalent sizes. Figure 8 illustrates the cascading failure processes in two such interdependent systems: the AS router network coupled with an ER network of equal size (Figure 8a) and the AS router network linked to an SF network of the same size (Figure 8b). As previously discussed, the system does not undergo a sudden collapse when the proportion of reinforced inter-layer links exceeds a critical threshold $\rho^{*}$. This finding offers insights into the design of more robust systems in practical applications.

## 5. Conclusions

This study establishes a general theoretical framework based on percolation, introduces a certain proportion of reinforced inter-layer links, and models a partially edge-coupled interdependent network containing reinforced inter-layer links. We first describe the topology and cascade failure process of the model in detail and then derive theoretical calculations of some critical parameters in the network based on bond percolation theory and probabilistic self-consistent equations, including the magnitudes of the finite and giant components—FCs and GCs—in the network, as well as the phase transition type cutoffs $p_{c}$. We then apply the proposed theoretical framework to artificially generated datasets of ER-ER and SF- SF networks, as well as real datasets for validation; we find that the theoretical predictions and simulation results are in good agreement. Moreover, we find that to prevent abrupt network transitions, a minimum fraction $\rho^{*}$ of reinforced inter-layer links exists, which serves as a boundary that distinguishes between networks where the giant component undergoes continuous and discontinuous phase transitions and those where the finite component undergoes discontinuous and transition-free states. Compared to the traditional reinforcement of nodes and the reinforcement of connected edges in interdependent networks, the reinforcement of dependency links used to eliminate sudden collapses requires fewer reinforcement resources. It is more effective as the coupling strength increases. Considering that the available reinforcement resources in the real world are limited, we will investigate the optimal allocation of reinforcement affiliation links in a future study.

## Data Availability

Data will be made available upon request to the corresponding author via email.

## Abbreviations

The following abbreviations are used in this manuscript:

GC	Giant component
FC	Functional component
EIN	Edge-coupled interdependent network
NIN	Node-coupled interdependent network
